# Comparison of *Echinococcus multilocularis* and *Echinococcus granulosus* hydatid fluid proteome provides molecular strategies for specialized host-parasite interactions

**DOI:** 10.18632/oncotarget.20761

**Published:** 2017-09-08

**Authors:** Chun-Seob Ahn, Jeong-Geun Kim, Xiumin Han, Insug Kang, Yoon Kong

**Affiliations:** ^1^ Department of Molecular Parasitology, Samsung Medical Center, Sungkyunkwan University School of Medicine, Suwon, Korea; ^2^ Qinghai Province Institute for Endemic Diseases Prevention and Control, Xining, China; ^3^ Clinical Research Institute for Hydatid Disease, Qinghai Provincial People’s Hospital, Xining, China; ^4^ Department of Molecular Biology and Biochemistry, Kyung Hee University School of Medicine, Seoul, Korea

**Keywords:** alveolar echinococcosis, cystic echinococcosis, hydatid fluid, proteome, protein-protein interaction networks

## Abstract

Alveolar and cystic echinococcoses, caused by the metacestodes of *Echinococcus multilocularis* and *E. granulosus*, are prevalent in several regions and invoke deleterious zoonotic helminthiases. Hydatid fluid (HF), which contains proteinaceous and non-proteinaceous secretions of the parasite- and host-derived components, critically affects the host-parasite interplay and disease progression. We conducted HF proteome profiling of fully mature *E. multilocularis* vesicle (nine months postinfection) and *E. granulosus* cyst (stage 2). We identified 120 and 153 proteins, respectively, in each fluid. Fifty-six and 84 proteins represented distinct species; 44 and 66 were parasites, and 12 and 18 were host-derived proteins. The five major parasite protein populations, which included antigen B isoforms, metabolic enzymes, proteases and inhibitors, extracellular matrix molecules (ECMs), and developmental proteins, were abundantly distributed in both fluids and also exclusively in one sample or the other. Carbohydrate-metabolizing enzymes were enriched in *E. granulosus* HF. In the *E. multilocularis* HF, proteins that constitute ECMs, which might facilitate adhesion and cytogenesis, were highly expressed. Those molecules had physical and functional relationships along with their biochemical properties through protein-protein interaction networks. Twelve host-derived proteins were largely segregated to serum components. The major proteins commonly and uniquely detected in these HFs and their symbiotic interactome relationships might reflect their biological roles in similar but distinct modes of maturation, invasion, and the longevity of the parasites in the hosts.

## INTRODUCTION

Echinococcosis refers to a disease complex caused by the metacestodes of the genus *Echinococcus*. More than seven *Echinococcus* spp., such as *E. granulosus* (G1), *E. ortleppi* (G4), *E. canadensis* (G6, 7, 8, and 10), *E. oligathus*, *E. vogeli*, and *E. multilocularis*, trigger human infections, among which *E. granulosus* and *E. multilocularis* are the most important pathogenic species [1]. Humans serve as intermediate hosts and are infected with the larval stage of the worms. When humans incidentally ingest parasite eggs, oncospheres hatched out from the eggs are activated in the small intestine. The oncospheres are released into the bloodstream and mostly end up lodged in the liver. The parasites grow into unilocular and multilocular cystic masses that result in cystic echinococcosis (CE) and alveolar echinococcosis (AE) [1].

CE is prevalent in nomadic areas in association with dog-rearing environments [2]. AE is increasingly detected in high-altitude forested and pastoral areas in the Northern Hemisphere, which includes enclaves in Europe, central Asia, and northwestern China [3, 4]. CE and AE are the most deleterious enzootic diseases and have a great impact on disability-adjusted life years [4, 5]. These larval cestodiases exemplify the top-ranking entities among the neglected tropical diseases by the World Health Organization due to their significant disease burden and associated socioeconomic losses (http://who.int/neglected_diseases/diseases/en/).

*Echinococcus granulosus* and *E. multilocularis* are the nearest phylogenetic neighbors in the family Taeniidae [6]: the two organisms share multiple aspects of growth and developmental plasticity and similarities in their intermediate and definitive hosts. The genomes of these two parasites each comprise approximately 115 megabases and show 96% sequence identity within the coding regions [7, 8]. They display the unique expansion of similar gene families that specialize in nutrient uptake from the host and protection from host defensive system [8]. Interestingly, they differ markedly in their biological features, such as morphological characteristics during growth and development, and invasion into adjacent tissues within the intermediate hosts. *E. granulosus* metacestode grows slowly to form unilocular cyst that expand and crowd the affected organs and tissues; conversely, *E. multilocularis* metacestode forms multivesiculated cystic mass and shows peripheral infiltration with central necrosis, which resembles invasive malignant tumors [1, 9]. The intermediate hosts of the parasites differ, ungulates and humans for CE and rodents and humans for AE.

The hydatid cyst has three major components: the germinal layer, the protoscolex, and hydatid fluid (HF). The germinal layer constitutes the outermost morphological contour and is responsible for the uptake of essential resources from the host through the syncytial membrane. The protoscolex asexually buds from the brood capsule, which protrudes from the germinal layer, and develops into an adult when it infects the definitive host [1, 5]. HF comprises numerous proteinaceous and non-proteinaceous materials, which are secreted from the parasites as well as absorbed from the host [10]. HF also harbors tegumental materials shed from the protoscolex and resources derived from metabolic turnover of the germinal layer [11, 12]. HF proteins are crucial at the host-parasite interface for maturation and survival of the parasites within the host and disease progression [12, 13]. The major biological functions of the HF proteins include activating and inhibiting immune and inflammatory cells and inducing Th2-biased host immune responses [11, 14]. The HF proteins participate in transportation and assimilation of host essential lipids, thus contributing to the long-term survival of the parasites [15]. The proteins also have protective functions that include antioxidant activity against oxidative stresses and proteolytic enzymes responsible for defense from host immune attacks [16, 17].

A number of studies have focused on identifying the global protein profile and biological reactivity of *E. granulosus* HF (EgHF) [11–13], but no study has yet analyzed the functional relationships and molecular interactions of EgHF proteins. The proteome profile of *E. multilocularis* HF (EmHF) was recently addressed during expressional analyses of *E. multilocularis* antigen B (EmAgB) isoforms [15]. However, comprehensive investigations and systematic analyses of EmHF regarding their biochemical and biological properties have not been conducted. More importantly, comparative data on *E. granulosus* and *E. multilocularis* HF proteomes are scarce. AE mass and CE cyst share a variety of biological similarities, but simultaneously, they show significant differences from each other. The HF from these closely related organisms might contain either unique or common proteins that are germane to their specialized biological features.

In the present study, we comparatively analyzed the proteome profiles of EmHF and EgHF obtained from experimentally or naturally infected hosts. HFs collected from immunologically competent hosts might reflect the genuine nature of HF proteomes [15]. Our data strongly suggest a possible biological function of HF proteins that could be correlated to the distinctive morphology, development, and longevity of parasites in hosts.

## RESULTS

### Proteome analysis of EmHF and EgHF grown in immunocompetent hosts

**Figure 1:**
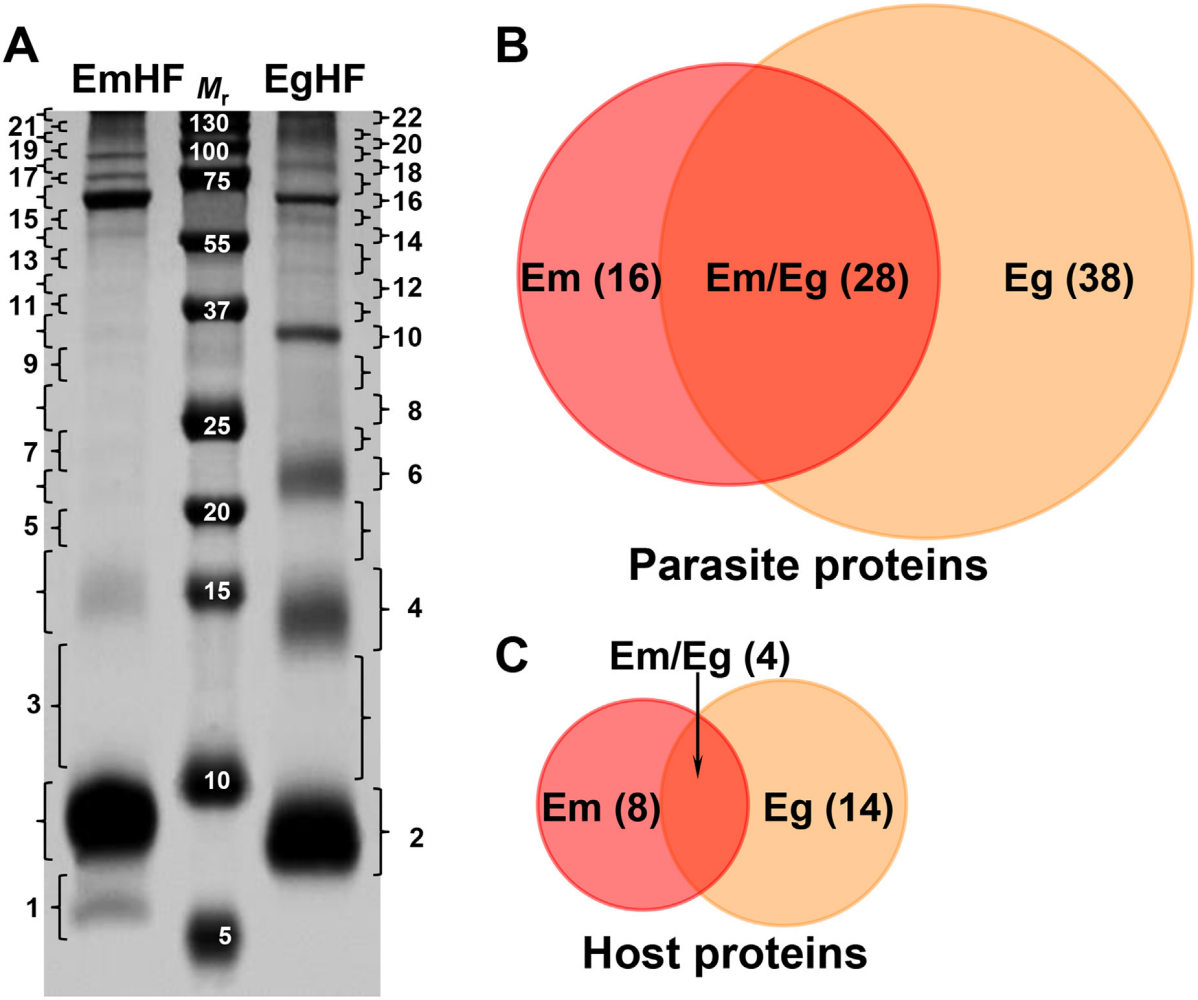
Electrophoretic profile and protein identification of the hydatid fluid of *E. multilocularis* (EmHF) and *E. granulosus* (EgHF) **(A)** EmHF and EgHF (each 20 μg) were separated by 10% Tricine SDS-PAGE under reducing conditions. The protein bands were divided into 22 pieces and processed with in-gel tryptic digestion prior to LC-ESI-MS/MS analysis. *M*_r_, molecular weight in kDa. **(B** and **C)** Comparison of protein components identified in the *Echinococcus* HF samples by Venn diagrams. Parasite proteins (B) and host-derived proteins (C).

Figure 1A displays the protein profile of fully mature EmHF (9 months) and EgHF (CE2 cyst) separated by 10% Tricine SDS-PAGE under reducing conditions. Comparative analyses of the both fluids revealed similar but distinct protein banding patterns. More than 50 protein bands, whose molecular weights ranged from below 6 kDa to over 200 kDa, were typically observed. The EmHF exhibited prominent bands at 6, 9, 16, and 64 kDa in addition to high molecular proteins > 72 kDa. The EgHF showed noticeable bands at 8, 14, 22, 34, and 64 kDa. High molecular proteins > 75 kDa were also deeply stained. In general, more prominent and clear banding patterns were evident in the EgHF compared with in the EmHF.

The protein bands were sliced into 22 gel pieces along with their molecular weights, tryptic digested, and analyzed through nano-liquid chromatography-electrospray ionization-multi-stage mass spectrometry (LC-ESI-MS/MS). We were able to identify 120 proteins from the EmHF, of which 85 (70.8%) and 35 proteins (29.2%) originated from the parasite and host, respectively. Of these proteins, 56 (46.7%) represented distinctive protein species; 44 parasite (36.7%) and 12 host proteins (10%) (Figures 1B and 1C, and Supplementary Table 1). The major protein fractions derived from the parasite constituted with 13 EmAgB isoforms (one EmAgB1, two EmAgB2, three EmAgB3a, four EmAgB3b/c, and three EmAgB4), nine N-acetylated α-linked acidic dipeptidase 2 (NAALAD2), each of six α2 macroglobulin and low-density lipoprotein receptor (LDLR), and three prosaposin (Table 1). We differentially classified EmAgB3 proteins as EmAgB3a and EmAgB3b/c because these molecules were synthesized from different genes: EmAgB3a from EmuJ_000381500 and EmAgB3b/c from EmuJ_000381600 and/or EmuJ_000381700 [17]. We also detected a number of ECM-associated proteins, such as different types of collagen α1 (IV and V), collagen XIα2, and fibrillar collagen FAp1α. Host proteins were largely allocated into serum components, specifically, albumin (15 entries) and immunoglobulin (three molecules) (Supplementary Table 1).

**Table 1:** Parasite proteins exclusively recognized in EmHF

Serial No.	Band nos.	Sum of emPAI^1^	Description	Accession no.^2^ (EmuJ_)	Mouse homolog^3^	Putative function
1	4	0.49	FABP	000550000	P04117	Transporter
2	4	0.49	Hedgehog	001200300	Q61488	Development
3	4	0.49	Zinc finger, C2H2	000194800	Q03267	Stress response
4	7	0.13	Collagen XI α2	000524200	Q64739	ECM
5	7	0.13	GST	000538300	P48774	Antioxidant
6	8	0.04	FGFR	000842900	Q9QWY8	Cellular interaction
7	10, 11, 15, 17-22	2.45	NAALAD2	000908900	Q9CZR2	Protease
8	10, 11, 13, 18, 19, 21, 22	0.99	α2 macroglobulin	000641100	Q6GQT1	Binding activity, Protease inhibitor
9	11	0.08	Annexin	000193700	P10107	Binding activity
10	12, 17, 18, 20-22	0.89	LDLR	000701800	O88572	Signaling
11	17	0.1	Gp50	001201600	-	Th2 response
12	17	0.1	CHP	001058700	-	Not-available
13	18	0.03	OTK	000212300	Q9QVP9	Kinase
14	19	0.02	Collagen α2(I)	000823800	Q64739	ECM
15	21	0.03	NAGAB	000716600	Q8BHN3	Carbohydrate metabolism
16	21, 22	0.11	Collagen α1(V)	000140100	O88207	ECM

^1^Exponentially modified protein abundance index.

^2^Accession numbers were obtained from NCBInr DB (http://www.ncbi.nlm.nih.gov/) and *E. multilocularis* DB (http://www.genedb.org/Homepage/Emultilocularis).

^3^Mouse homologs were retrieved using Uniprot software (http://www.uniprot.org/).

CHP, conserved hypothetical protein; FABP, fatty acid binding protein; FGFR, fibroblastic growth factor receptor; GST, glutathione transferase; Gp50, diagnostic antigen gp50; LDLR, low density lipoprotein receptor; NAALAD2, N-acetylated α-linked acidic dipeptidase 2; NAGAB, neutral α glucosidase AB; OTK, tyrosine protein kinase otk.

We detected 153 proteins (132 parasite [86.3%] and 21 host proteins [13.7%]), among which 84 entities (54.9%) were distinct proteins (66 parasite [43.1%] and 18 host proteins [11.8%]) in the EgHF. A total of 42 entries of diverse EgAgB isoforms comprised 11 each of EgAgB1 and EgAgB2, six EgAgB3, and 14 EgAgB4. In addition, eight glycoprotein antigen 5, five peptidase inhibitor 16, and four gynecophoral canal proteins (fasciclin) [18] consisted of the major parasite protein fractions (Table 2). We found abundant serum components including serotransferrin and albumin as host-derived molecules (Supplementary Table 2).

**Table 2:** Parasite proteins observed uniquely in EgHF

Serial No.	Band nos.	Sum of emPAI^1^	Description	Accession no.^2^ (EgrG_)	Mouse homolog^3^	Principal functions
1	3-5	0.63	ECP	000378300	-	Not-available
2	3	0.28	ECP	000956500	-	Not-available
3	3	0.22	SOD1	000638300	P08228	Antioxidant
4	3	0.26	Profilin	000122100	-	Actin binding
5	4	1.86	ECP	000079500	-	Not-available
6	5, 6	1.62	Ferritin	000382200	P09528	Iron transport
7	5	0.2	snRNP	000525910	-	Isomerase
8	6	2.08	ECP	000596300	-	Not-available
9	6	1.29	ECP	000315600	-	Not-available
10	6	0.35	ThioTrx	000791700	P35700	Redox homeostasis
11	6-10	1.42	PI16	000766600	P49935	Protease inhibitor
12	7	0.16	Niemann Pick	000682900	Q9Z0J0	Lipid metabolism
13	7	0.13	Complement TNF	001189200	Q8R2Z0	Carbohydrate metabolism
14	7	0.12	TPI	000416400	P17751	Carbohydrate metabolism
15	8	0.57	14-3-3 protein	000789700	P16054	Signaling
16	8	0.26	IoMT	001133400	P23506	Transferase
17	8	0.1	PGM	000799500	O70250	Carbohydrate metabolism
18	10, 11	1.52	MDH	001185000	P08249	Carbohydrate metabolism
19	10	0.31	LDHA	000660800	P06151	Carbohydrate metabolism
20	10, 11	0.4	Succinyl CoA syn	001199000	Q9Z2I8	Ligase
21	10	0.23	IGBP	000799300	P05017	Signaling
22	10	0.29	Transaldolase	000092800	Q93092	Carbohydrate metabolism
23	10	0.08	PDH	000956200	O70571	Carbohydrate metabolism
24	12	1.15	OA	001032200	P29758	Transferase
25	13	1.54	Citrate synthase	001028500	Q9CZU6	Carbohydrate metabolism
26	13	0.4	NADPH	001068500	P54071	Carbohydrate metabolism
27	13	0.25	PDHE1	000590700	P35486	Carbohydrate metabolism
28	13	0.12	Acyl CoA HT	001087900	Q9DBK0	Lipid metabolism
29	14, 15	1.21	Calnexin	000875100	P35564	Chaperone
30	14	0.27	Iron:zinc AP	001169400	Q8BX37	Hydrolase
31	14	0.12	GD	000589100	P26443	Oxidoreductase
32	16	1.33	VAT	000317300	Q8BRU6	Transport
33	16	0.06	Lysyl oxidase	000217900	P58022	Oxidoreductase
34	16	0.12	AspRNA	000777100	Q922B2	Protein biosynthesis
35	17, 19	1.21	PEPCK	000292700	Q8BH04	Carbohydrate metabolism
36	20	0.07	Macroglobulin	000641200	Q3UU35	Serine protease inhibitor
37	21	0.05	Collagen α1(XV)	000729300	O35206	ECM
38	22	0.04	Hemicentin 1	000422350	D3YXG0	Cell cycle

^1^Exponentially modified protein abundance index.

^2^Accession numbers were obtained from NCBInr DB (http://www.ncbi.nlm.nih.gov/) and *E. granulosus* DB (http://www.genedb.org/Homepage/Egranulosus).

^3^Mouse homologs were retrieved using Uniprot software (http://www.uniprot.org/).

Acyl CoA HT, acyl coenzyme A hydrolase transferase; AspRNA, aspartyl tRNA synthetase cytoplasmic; Complement TNF, complement C1q tumor necrosis factor; ECP, expressed conserved protein; FABP, fatty acid binding protein; FGFR, fibroblastic growth factor receptor; GD, glutamate dehydrogenase; GST, glutathione transferase; IoMT, protein l isoaspartate o methyltransferase; Iron:zinc AP, iron:zinc purple acid phosphatase; IGBP, insulin growth factor binding protein; LDHA, lactate dehydrogenase A; LDLR, low density lipoprotein receptor protein; MDH, malate dehydrogenase (mitochondrial); NAALAD2, N-acetylated alpha-linked acidic dipeptidase 2; NADPH, NADPH dependent isocitrate dehydrogenase; Niemann-Pick, Niemann Pick C2 protein; OA, ornithine aminotransferase; PDH, pyruvate dehydrogenase; PDHE1, PD E1 component subunit; PEPCK, phosphoenolpyruvate carboxykinase; PGM, phosphoglycerate mutase; PI16, peptidase inhibitor 16; snRNP, U snRNP cyclophilin; SOD1, superoxide dismutase 1 (soluble); Succinyl CoA syn, succinyl coenzyme A synthetase α subunit; TPI, triosephosphate isomerase; ThioTrx, thioredoxin peroxidase; VAT, vesicular amine transporter.

Overall, LC-MS/MS analysis identified 82 parasite proteins, with 16 EmHF proteins, 38 EgHF proteins, and 28 proteins shared between EmHF and EgHF (Figure 1B, and Tables 1-3). We identified 26 different host-derived proteins in the EmHF and EgHF; four proteins were common to both fluids, whereas we detected eight only in EmHF and 14 proteins exclusively in EgHF (Figure 1C, and Supplementary Table 3 and Supplementary Table 4). These results suggested the close phylogenetic and evolutionary relationships but also distinct biological behaviors of these two parasites within their host environments.

**Table 3:** Parasite proteins identified in both EgHF and EmHF

Serial No.	Band nos.	Sum of emPAI^1^	Description	Accession no.^2^ (EmuJ_/EgrG_)	Mouse homolog^3^	Principal functions
1	2	3.51	EmAgB1	000381200	-	Lipid binding, Th2 response
	2-11, 18	44.68	EgAgB1	000381200	-	
2	2, 3	11.55	EmAgB2	000381100	-	Lipid binding, Th2 response
	2-10, 13, 15	16.11	EgAgB2	000381100	-	
3	2-4	29.49	EmAgB3a	000381500	-	Lipid binding, Th2 response
4	1-4	27.34	EmAgB3b/c	000381600/700	-	Lipid binding,
	2-6, 9	12.86	EgAgB3	000381600	-	Th2 response
5	2-4	5.67	EmAgB4	000381400	-	Lipid binding, Th2 response
	2-12, 14, 16, 18	54.24	EgAgB4	000381400	-	
6	22	0.08	Collagen α1(IV)	000140000	P02463	ECM
	22	0.08		000144350		
7	6, 15	0.13	GP5	000184900	P21845	Serine protease, Th2 response
	5, 6, 10, 11, 13, 14, 16, 18	20.24		000184900		
8	9, 10	0.22	Cystatin	000849600	P21460	Protease inhibitor
	9, 10	1.36		000849600		
9	10	0.19	MDH	000417100	P14152	Carbohydrate metabolism
	10	0.68				
10	10, 11	0.18	GAPDH	000254600	Q64467	Carbohydrate metabolism
	10	0.55		000254600		
11	11, 12	0.57	FBA	000905600	Q91Y97	Carbohydrate metabolism
	10-12	1.86		000905600		
12	11, 12	0.44	Cathepsin B	000790200	Q923E4	Cysteine protease
	11, 12	1.69		000790200		
13	11, 12, 19	0.1	Laminin	000068100	Q61292	ECM
	22	0.13		000068100		
14	13	0.5	Enolase	000514200	P17182	Carbohydrate metabolism
	12	0.41		000514200		
15	14	0.52	EP45	000824000	O08797	Cysteine protease
	12	0.54		000824000		
16	16	0.14	GCP	000824400	Q9JK48	Binding activity
	15, 17	1.07		000824400		
17	18	0.63	GCP	000712600	Q9JK48	Binding activity
	20, 21	1.89		000712600		
18	19	0.03	α glucosidase	000141000	P70699	Carbohydrate metabolism
	21	0.1		000143500		
19	17, 21, 22	0.25	HSPG	000575900	Q05793	Binding activity
	21	0.31		000575900		
20	18	0.08	AAO	000530400	Q8JZQ5	Oxidoreductase
	17, 19	0.39		000530400		
21	18	0.04	ADAMTS3	000969100	E9Q287	Protease
	17, 18	0.47		000969100		
22	18, 21, 22	0.12	Prosaposin	000733100	Q61207	Signaling, growth
	21	0.15		000733100		
23	21	0.07	α mannosidase	000704400	O09159	Carbohydrate metabolism
	18	0.37		000704400		
24	22	0.04	Peroxidasin	000733600	Q3UQ28	Peroxidase
	21	0.09		000733600		
25	22	0.06	Emb9	000139900	P09055	Binding activity
	22	0.08		000144400		
26	22	0.11	Notch	000343000	Q01705	Binding activity
	22	0.2		000343000		
27	22	0.11	EGF	000255800	E9Q2T3	Adhesion
	22	0.23		000255800		
28	15	0.08	PGI	000626300	P06745	Carbohydrate metabolism
	15	0.68		000626300		

^1^Exponentially modified protein abundance index.

^2^Accession numbers were obtained from NCBInr DB (http://www.ncbi.nlm.nih.gov/) and *E. multilocularis* and *E. granulosus* DB (http://www.genedb.org/Homepage/).

^3^Mouse homologs were retrieved using Uniprot software (http://www.uniprot.org/).

AAO, amiloride sensitive amine oxidase; ADAMTS3, ADAMTS protein 3; EgAgB, *E. granulosus* antigen B; EmAgB, *E. multilocularis* antigen B; Emb9, abnormal EMBroylocus tagsis emb 9; EP45, estrogen regulated protein EP45; FBA, fructose bisphosphate aldolase; GAPDH, glyceraldehyde 3-phosphate dehydrogenase; GCP, gynecophoral canal protein; GP5, glycoprotein antigen 5; HSPG, basement membrane specific heparan sulfate; MDH, malate dehydrogenase (cytosolic); PGI, phosphoglucose isomerase.

### Gene ontology (GO) terms assigned to proteins identified in EmHF and EgHF

**Figure 2:**
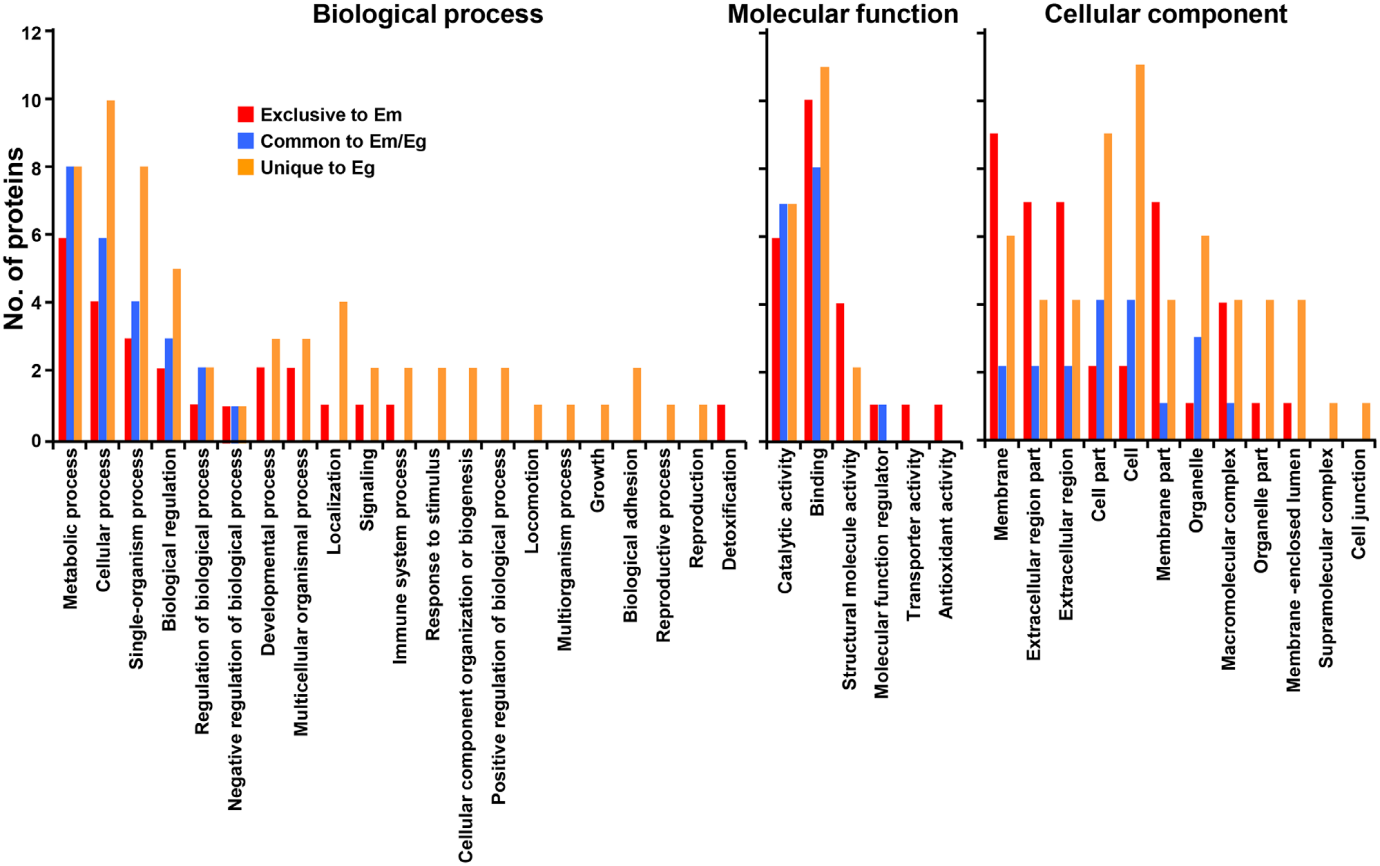
Classification of gene ontology of parasite proteins identified from *E. multilocularis* hydatid fluid (EmHF) and *E. granulosus* HF (EgHF) The number of identified proteins in each functional group is shown in the histogram. The terms associated with the biological process, molecular function, and cellular component were adopted from Blast2GO based on similarity patterns using the second level of the GO hierarchy [19].

The systematic analysis employing GO terms were assigned to the identified proteins on the basis of similarity patterns by Blast2GO in the second level subcategories [19]. Figure 2 presents the distribution patterns of the parasite proteins along with their given ontological terms. The proteins associated with biological process revealed somewhat dissimilar distribution patterns. Molecules related to metabolic process, cellular process, single-organism process, and biological regulatory molecules were commonly detected in both fluids. We also recognized proteins involved in developmental and multicellular organism processes, localization, and signaling in both samples. However, molecules associated with the immune and protective system, biogenesis, and adhesion were observed exclusively in EgHF. In the molecular function and cellular component subcategories, relatively large proportions of the proteins were shared by both parasites, but those related to molecular function were more prominent in EmHF and those connected to cellular compartment were more enriched in EgHF.

**Figure 3:**
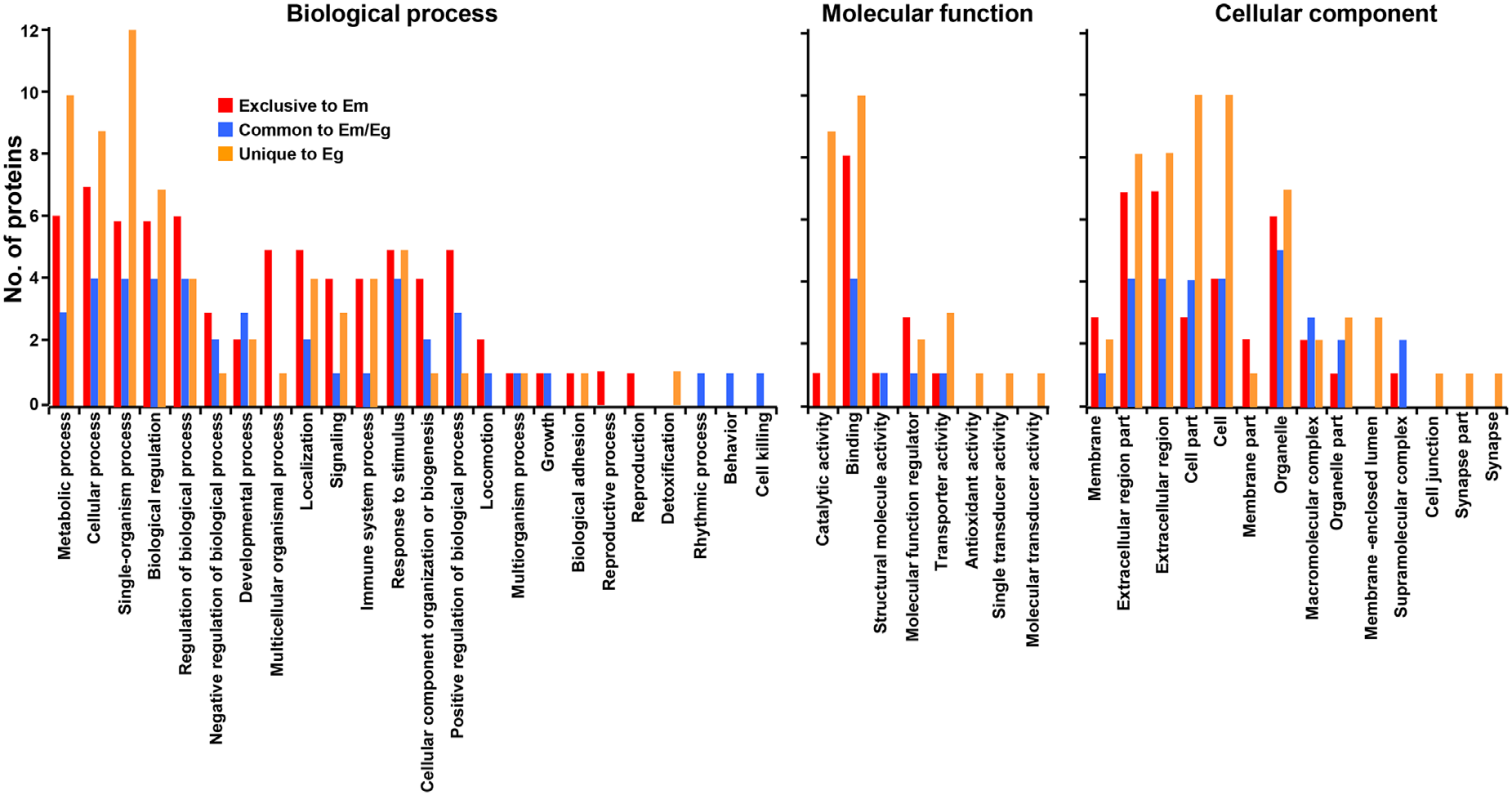
Analysis of gene ontology of host-derived proteins recognized in *E. multilocularis* hydatid fluid (EmHF) and *E. granulosus* HF (EgHF) The number of proteins in each functional group is shown by histogram. The terms assigned to the biological process, molecular function, and cellular component were tailored from Blast2GO based on similarities employing the second level of the GO hierarchy [19].

Host proteins commonly and uniquely detected were largely grouped into metabolic process, cellular process, single-organism process, biological regulator and stimulus responses (biological process), and binders and catalysts (molecular function). The proteins linked to cellular component exhibited dissimilar distribution patterns along with the respective HF. Molecules located in membrane, extracellular regions, and organelle were relatively abundant in EmHF, whereas those associated with cellular and organelle structure were largely distributed in EgHF (Figure 3).

### Functional characterization of identified proteins

**Figure 4:**
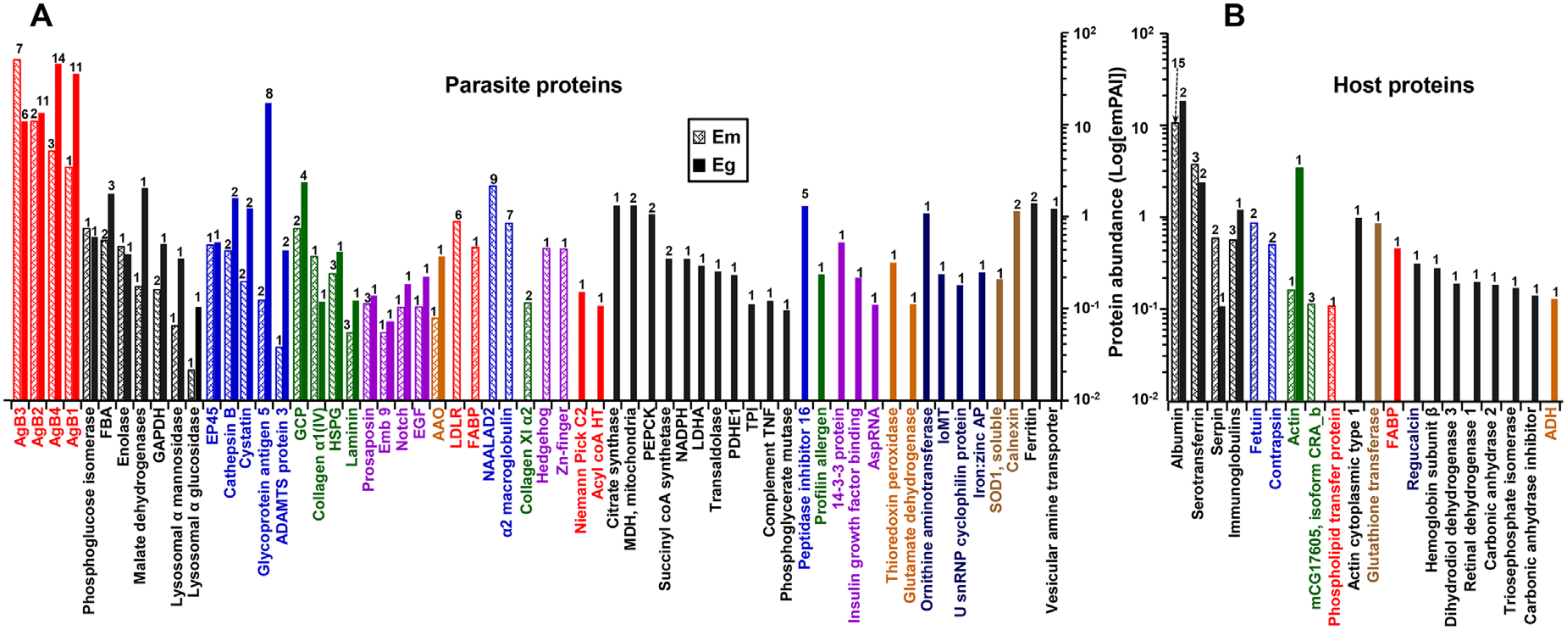
Differential heat map of the *E. multilocularis* hydatid fluid (EmHF) and *E. granulosus* HF (EgHF) proteins The 26 most abundant parasite proteins **(A)** and top five host proteins **(B)** that showed emPAI values above 0.01 were analyzed. Protein abundance is presented by log[emPAI]. Antigen B isoforms, whose main functions are related to lipid transportation and assimilation, and Th2 responses are marked by red. Enzymes involved in carbohydrate metabolism are indicated by black. Molecules associated with proteolysis and inhibitors are shown in blue. Extracellular matrix and cytogenetic proteins are denoted by green. Molecules associated with cell growth and development are shown in purple. AgBs, Em and Eg antigen Bs; Ag5, glycoprotein antigen 5; AspRNA, aspartyl tRNA synthetase (cytoplasmic); GAPDH, glyceraldehyde 3-phosphate dehydrogenase; FBA, fructose bisphosphate aldolase; EP45, estrogen regulated protein 45; HSPG, basement membrane specific heparan sulfate; Emb9, abnormal EMBroylocus tagsis(embryogenesis family member) emb 9; Notch, neurogenic locus notch protein; EGF, EGF domain protein.

The proteins identified in the EmHF and EgHF were further analyzed in accordance with their principal biochemical properties. We selected proteins that showed high abundance by emPAI values greater than 0.01 to ensure confidence in our protein identification and reproducibility. As shown in Figure 4A and Table 3, five major protein fractions were abundant in both fluids: i) several isoforms of antigen B molecules (AgB1-AgB4); ii) metabolic enzymes (phosphoglucose isomerase, fructose bisphosphate aldolase, enolase, malate dehydrogenase, glyceraldehyde 3-phosphate dehydrogenase, and lysosomal α mannosidase); iii) proteases and inhibitors (estrogen regulated protein 45, cathepsin B, glycoprotein antigen 5, ADAMTS protein 3, and cystatin); iv) molecules associated with ECM and cytogenesis (gynecophoral canal protein, collagen, basement membrane-specific heparan sulfate, and laminin); and v) developmental and signaling proteins (abnormal EMBryolocus tagsis [embryogenesis family member] emb 9, neurogenic locus notch, and EGF domain protein).

N-acetylated α-linked acidic dipeptidase-like 2 (NAALADL2), which might be involved in adhesion and invasion through its proteolytic activity, and α2 macroglobulin, which inhibits protease activity, were uniquely and abundantly expressed in EmHF. We observed a signal-related molecule of LDLR only in EmHF (Table 1). In contrast, diverse enzymes involved in carbohydrate metabolism (citrate synthase, malate dehydrogenase, phosphoenolpyruvate carboxykinase, succinyl CoA synthetase, nicotineamide adenine dinucleotide phosphate reduced form, phosphopyuvate dehydrogenase E1, triosephosphate isomerase, and phosphoglycerate mutase) were exclusively detected in EgHF. We recognized antioxidant proteins (superoxide dismutase and thioredoxin peroxidase) and chaperone molecule (calnexin). In addition, signaling molecules (14-3-3 proteins and insulin-like growth factor-binding protein), ornithine aminotransferase, peptidase inhibitor 16, and three expressed conserved proteins (EgrG_000596300, EgrG_000079500, and EgrG_000315600) constituted relatively large fractions in EgHF (Table 2).

Major fractions of the host-derived molecules commonly found in both fluids were serum components, which included albumin, serotransferrin, α1 protease inhibitor (serpin), and immunoglobulins. We detected lipid- and carbohydrate-metabolizing enzymes exclusively in the EgHF proteome (Figure 4B, Supplementary Table 3 and Supplementary Table 4).

### Integrated network for protein-protein interactions (PPIs) in the HF proteomes

Protein-protein interactions are critically involved in most cellular and biochemical processes. During proteome analysis of the EmHF and EgHF, we identified key molecules engaged in cytoskeletal and cytogenesis in EmHF, which included laminin, integrin, and a subset of diverse collagens. We also found considerable numbers of hub proteins, which importantly participated in carbohydrate metabolism in the EgHF (malate dehydrogenase 2, lactate dehydrogenase, enolase, transaldolase, and triosephosphate isomerase) (Tables 1-3). Table 4 summarizes the numbers and functionality of the interacting proteins in the EmHF and EgHF identified through the STRING ver10 algorithm.

**Table 4:** Functional categorization of interacting proteins identified in EgHF and EmHF

Interaction properties	No. of proteins found in EgHF	No. proteins of identified in EmHF
Carbohydrate metabolism	27	7
Adhesion and cytogenesis	18	20

**Figure 5:**
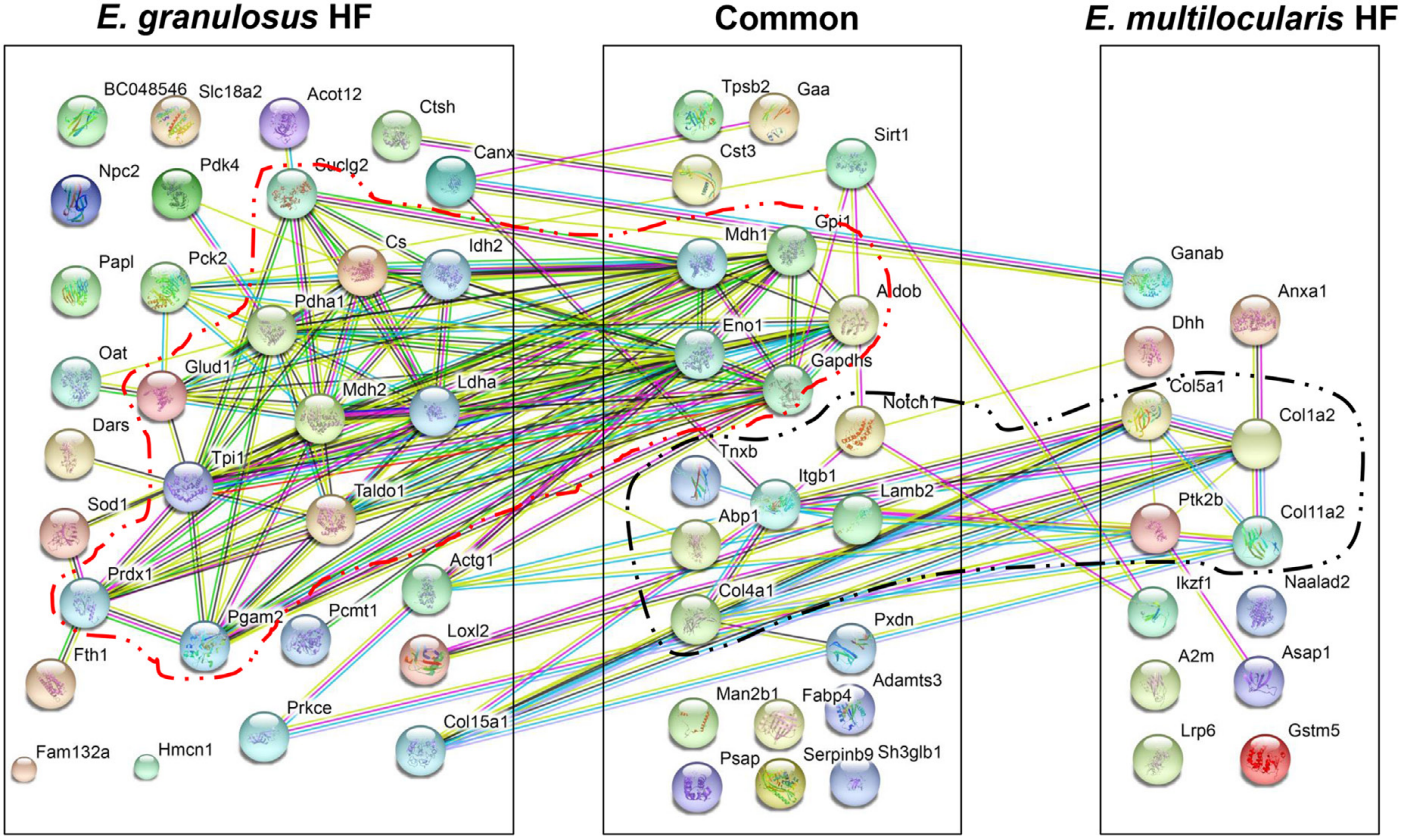
Protein-protein interaction networks in *E. multilocularis* hydatid fluid (EmHF) and *E. granulosus* HF (EgHF) The correlated interactions extracted from the EmHF and EgHF proteins are presented with their predicted functional partners by STRING ver10.0 (http://string-db.org/). The interactions are shown in confidence view. Proteins marked within lines represent a robust association. A2m, α2 macroglobulin; Abp1, amiloride sensitive amine oxidase; Acot12, acyl-CoA thioesterase 12; Actg1, γ actin (cytoplasmic 1); Adamts3, ADAM metallopeptidase; Aldob, fructose-bisphosphate aldolase B; Anxa1, annexin A1; Asap1, ankyrin repeat and PH domain1; BC048546, cDNA sequence BC048546; Canx, calnexin; Col11a2, collagen type XI α2; Col15a1, collagen type XV α1; Col1a2, fibrillary collagen type I α2; Col4a1, collagen type IV α1; Col5a1, collagen type V α1; Cs, citrate synthase; Cst3, cystatin C; Ctsh, cathepsin H; Dars, aspartyl-tRNA synthetase; Dhh, desert hedgehog; Eno1, enolase 1; Fabp2, fatty acid binding protein 2; Fam132a, family with sequence similarity 132 (member A); Fth1, ferritin heavy chain 1; Gaa, α-glucosidase; Ganab, glucosidase 2α neutral subunit; Gapdhs, glyceraldehyde-3-phosphate dehydrogenase, Glud1, glutamate dehydrogenase 1; Gpi1, glucose phosphate isomerase 1; Gstm5, glutathione transferase (mu-class); Hmcn1, hemicentin 1; Idh2, isocitrate dehydrogenase 2; Ikzf1, zinc finger 1; Itgb1, integrin β1; Lamb2, laminin β2; Ldha, lactate dehydrogenase A; Loxl2, lysyl oxidase 2; Lrp6, low density lipoprotein receptor-related protein 6; Man2b1, mannosidase 2 αB1; Mdh1, malate dehydrogenase 1; Mdh2, malate dehydrogenase 2 (mitochondrial); Naalad2, N-acetylated α-linked acidic dipeptidase 2; Notch1, neurogenic locus notch protein; Npc2, Niemann Pick (C2); Oat, ornithine aminotransferase; Papl, RIKEN cDNA C330005M16; Pck2, phosphoenolpyruvate carboxykinase 2 (mitochondrial); Pcmt1, isoaspartate o methyltransferase 1; Pdha1, pyruvate dehydrogenase E1 α1; Pdk4, pyruvate dehydrogenase kinase; Pgam2, phosphoglycerate mutase 2; Prdx1, peroxiredoxin 1; Prkce, protein kinase C; Psap, prosaposin; Ptk2b, protein tyrosine kinase 2; Pxdn, peroxidasin; Serpinb9, serpin family B member 9 protease inhibitor; Sh3glb1, SH3 domain containing GRB2-like endophilin B1; Sirt1, NAD-dependent deacetylase sirtuin-1; Slc18a2, solute carrier family 18 (vesicular monoamine); Sod1, superoxide dismutase 1; Suclg2, succinate-CoA ligase (GDP-forming) subunit β (mitochondrial); Taldo1, transaldolase 1; Tnxb, tenascin XB; Tpi1, triosephosphate isomerase 1; Tpsb2, tryptase β2.

We analyzed the topology and centrality of the PPI network in the EmHF and EgHF to elucidate functional protein organization. Figure 5 depicts the integrated PPI network of the identified proteins. Numerous molecules interacted with one or more other proteins. One each of major functional cluster that showed a strong PPI relationship was evident along with their biochemical properties and/or biological roles. Of 66 proteins identified in the EgHF, 45 (68.2%) revealed a complicated interaction network in which 24 (36.4%) were tightly associated with carbohydrate metabolism (confined within the red dotted circle). Of 35 proteins in the EmHF, 24 (68.6%) displayed interactome relationships. Molecules associated with ECM, adhesion, and cytogenesis (10 species; 28.6%) demonstrated a strong functional network (Figure 5, marked by black dotted circle). The number of interacting proteins appeared to be larger than that of annotated proteins due to additional functionality of each molecule.

## DISCUSSION

AE and CE metacestodes thrive for considerable periods in immunologically competent hosts and invoke significant illness. HF proteins continuously interact with the host as a first-line effector system to confront cytopathic environments [12-15, 20]. Their molecular interactions might critically affect parasite survival and subsequent disease progression. In this study, we comparatively compiled proteome profile for AE and CE HF. The HFs shared biochemical and biological similarities. We identified 120 and 153 proteins from fully matured AE and CE HF, respectively, in which 56 and 84 proteins represented distinctive species. Those proteins were largely parasite proteins (44 and 66 molecules); host-derived proteins constituted minor fractions (12 and 18 molecules).

AE and CE metacestodes develop in intermediate hosts with different growth rates and morphological characteristics. The AE metacestode grows more rapidly than the *E. granulosus* metacestode. Morphologically, the AE metacestode typically forms a multilocular vesicular mass, while the CE metacestode matures into a large unilocular cyst [1]. The major proteins detected in those HFs and their functional PPI networks suggest both common and differing biological activity specific to the respective parasites’ characteristic modes of maturation within hosts.

Diverse antigen B isoforms were widely distributed in both fluids. In EmHF, EmAgB3 constituted the most enriched population, followed by EmAgB2, EmAgB4, and EmAgB1. Conversely, the fractions of EgAgB isoforms were reversed, with major fractions of EgAgB1 and EgAgB4 and a minor EgAgB3 fraction (Tables 1 and 2, Figure 4). We could not detect EmAgB5 or EgAgB5 from fully mature EmHF or CE2 HF. Expression of these molecules might be associated with premature adult stages [21]. Antigen B proteins are multifunctional lipoproteins that inhibit neutrophil chemotaxis and dendritic cell maturation and the induction of host immune responses [14, 22, 23]. These molecules also function as hydrophobic-ligand binding proteins [15] and constitute the major components to supply energy and resources for maintaining cellular viability by providing essential host lipids [24] because cestode parasites lack the enzymes involved in lipid biosynthesis [7, 8]. The dissimilar expression patterns of several antigen B molecules, which revealed interspecies differences, suggest their idiosyncratic roles in host-parasite specific interactions and pathobiological changes during the course of disease progression [11, 15].

The second most abundant proteins were carbohydrate-metabolizing enzymes. These molecules did not bear signal peptide, but significant amounts appeared to be secreted. The enzymes were commonly found in both fluids, and some additional molecules were detected in the EgHF. Carbohydrate-metabolizing enzymes are repeatedly observed in the secretome of several helminths including *E. granulosus* [11, 13]. We thought that these molecules might be excreted through specific mechanism, i.e., extracellular vesicle mediated secretion rather than by passive leakage via tegumental shedding and/or turnover [25, 26]. Previous studies indicated that some carbohydrate-metabolizing enzymes exerted moonlighting effects, such as the antioxidant detoxification and glutathione redox cycle, cellular adhesion and invasion, plasmin-mediate proteolysis, and IgA immune responses, together with their primary biochemical roles [27–30]. More enzymes in the EgHF might be correlated with longer survival of CE than AE owing to the more sufficient energy sources and by protecting the parasite from host defensive system.

Proteolytic enzymes and their inhibitors play major roles in numerous cellular events [31, 32]. We detected relatively large amounts of these molecules, which strongly suggest that vigorous molecular biochemical and cell biological events operate dynamically in HFs. It is notable that we detected a great deal of NAALAD2 in EmHF. Upregulation of NAALAD2 expression has been associated with exacerbation of cancerous lesions and facilitation of tumor invasion [33]. Interestingly, we detected active NAALAD2 transcription only in the early developmental stages, one to three months, but not later than six month postinfection (Supplementary Figure 1). We also did not detect the NAALAD2 transcript in the CE2 stage. The considerable amounts of NAALAD2 observed in fully mature EmHF indicated the continuous accumulation of previously synthesized molecules (Table 2). This result suggests that infiltrative growth of AE vesicle might be associated with early but not late stage of infection. In the late stage, AE vesicle might expand by the inward growth of numerous daughter vesicles containing protoscolices, similar to the expansion of CE cyst. NAALAD2 protein accumulated in the EmHF might contribute to peripheral infiltrative growth to some extent. At the same time, highly crowded daughter vesicles increased unfavorable anoxic internal microenvironments and nutritional imbalance, which might result in the characteristic AE pathology of central necrosis. Further studies will be needed to gain deeper insight into the functional relevance and relationships between transcriptional activity and proteolytic activity, and infiltrative growth of AE vesicle.

Both HFs harbored some molecules closely related to developmental processes, i.e., notch, prosaposin, emb 9, hedgehog, and EGF domain proteins. They might participate in differentiation of neoblasts or stem cells during early embryogenesis and protoscolex maturation [34, 35]. We also detected tissue connection and membrane structural proteins, such as different types of collagen chains, laminin, integrin, and HSPG (Figure 4). These ECM-associated molecules might exert their effects during adhesion, cytogenesis, and formation of cellular architecture through interactions with other molecules [36].

Host proteins comprised approximately 10% of the total proteins; this proportion was similar to that of *Taenia solium* metacestode cyst fluid [37]. Many of the host proteins detected in both HFs were serum proteins, strongly supporting the notion that *Echinococcus* might take up and exploit these substances. Other host molecules that function in the immune modulation and protective activity found in HF might also be absorbed through the highly permeable germinal layer during host-parasite interactions [12, 38].

We either did not detect some previously characterized proteins whose functions might be relevant to parasite homeostasis or they were exclusive to only one sample. For example, we observed membrane-sensing endophilin B1 (previously known as P29) and signal-related 14-3-3 proteins only in EgHF (Figure 4 and Table 2). However, the authentic activity of those molecules was confirmed in *E. multilocularis* [39, 40]. This apparent discrepancy could suggest that expression levels of these molecules in *E. multilocularis* metacestode might not be sufficiently high to have been detectable under our experimental conditions. The use of different technical systems might also explain the detection of different protein repertories. Alternatively, the expression patterns of these proteins might be differentially regulated in different host conditions. A previous study demonstrated that AE vesicles grown in immunocompetent mice expressed significantly low levels of 14-3-3 protein compared with those grown in immune deficient nude mice [41].

Protein-protein interaction networks may provide valuable information on integrated protein functions [42]. One major PPI network was evident in each EmHF and EgHF, which might be correlated with survival strategies of these parasites (Figure 5). In EmHF, interactions among ECM-associated molecules revealed a symbiotic cluster. The hub proteins (integrin, laminin, and protein kinase 2) mediate cellular adhesion and cytogenesis, which might reflect morphological features characteristic of AE. AE might express more ECM-associated molecules to construct and maintain the highly complicated multilocular architecture [1]. The main PPI network found in EgHF was related to carbohydrate metabolic pathways in which lactate dehydrogenase, transaldolase 1, and enolase 1 were the central proteins. Because CE cyst grows to be considerably large and shows longer lifespans than AE vesicle, enzymes and pathways involved in carbohydrate metabolism might be more active in CE cyst.

The main characteristics that differentiate these two phylogenetically closed parasite species are the modes of proliferation and invasion in the hosts. The protoscolex and germinal layer should have diverse bioactive molecules that exert their activity during the parasites’ maturation. Therefore, a parallel proteomic analysis on the germinal layer and the protoscolex would provide more direct evidence of their pathogenesis. This should form the basis for future investigations.

In conclusion, this study comparatively analyzed the LC-MS/MS based proteome profiles of fully mature HF from *E. multilocularis* and *E. granulosus* metacestodes grown in immunocompetent hosts. We identified 120 and 153 proteins, respectively, from the EmHF and EgHF, most of which had parasitic origins. The major parasite protein fractions including multifunctional antigen B isoforms, carbohydrate-metabolizing enzymes, proteolytic enzymes and inhibitors, and ECM molecules were either common to both HFs or exclusive to one sample. Functional PPI networks established for the first time indicated that at least each one of major network might operate in both EmHF (symbiotic interactome relationship between EMC molecules) and EgHF (carbohydrate metabolism), which might reflect similar but different biological behaviors during the worm’s maturation. Our results also highlight future strategies for developing chemotherapeutic and vaccine modalities against fatal chronic communicable diseases. Blocking antigen B isoforms may impede the uptake and transport of essential host lipids, which could significantly affect parasite survival. Disrupting key bioactive molecules involved in carbohydrate metabolism and ECM interactions may also be useful for novel interventions.

## MATERIALS AND METHODS

### Parasites and sample preparation

Kunming mice were each infected intraperitoneally with 1000 viable *E. multilocularis* protoscolices (EmRUS12 strain) [43] collected from naturally infected voles (*Microtus fuscus*) in an AE endemic area (Dari County, Qinghai Province, China) [24]. Metacestode vesicles grown for 9 months were harvested from the liver surface and peritoneal cavity. The vesicles were carefully decorticated and washed more than 10 times with physiological saline. The EmHF was aseptically drawn from individual intact cysts using a 26-gauge needle and were pooled in the presence of protease inhibitor cocktail (25 ml per tablet; Roche, Mannheim, Germany). In addition, AE masses were chronologically collected from the mice at 1, 3, 6, 9, and 14 months postinfection and were immediately used for RNA extraction using a commercially available kit (iNtRON, Seongnam, Korea). *E. granulosus* metacestode cyst (sensu stricto, G1 genotype) was obtained from a naturally infected sheep in a local abattoir in Xining (Qinghai Province, China). EgHF was drawn from a single fertile hepatic CE2 cyst [11]. The germinal layer and protoscoleces were used for RNA preparation. Both HFs were centrifuged at 500 g for 2 min, followed by 20000 g for 1 h. Supernatants (EmHF and EgHF) were dialyzed against phosphate buffered saline (PBS, 100 mM, pH 7.4) for 4 h, concentrated by lyophilization, and stored at -80°C. All procedures during the worm collection and HF dialysis were done at 4°C unless otherwise specified.

All protocols including parasite infection, recovery of the worms under intraperitoneal injection of 500 μl of a 7:3 mixture of alfaxlone (10 mg/ml) and xylazine (23.32 mg/ml) and euthanasia of the moribund mice were approved by the Institutional Review Committee of the Qinghai Province Institute for Endemic Disease Prevention and Control (protocol 2013-7-22).

### Protein identification

EmHF and EgHF (each 20 μg) were separated by Tricine SDS-PAGE (10%) under reducing conditions. The gels were stained with Coomassie Brilliant blue G-250 (CBB). Protein bands were divided into 22 gel pieces along with their CBB-stained banding patterns by their molecular weights and were sliced into fragments. Disulfide bonds were reduced with 10 mM dithiothreitol. The proteins were alkylated with iodoacetamide (10 mg/ml) in 100 mM NH_4_HCO_3_. After dehydration and rehydration, the proteins were trypsin-digested (12.5 ng/μl) and the peptides were extracted from the gel pieces using a 50% acetonitrile solution that contained 5% formic acid. All chemicals were purchased from Sigma-Aldrich (St. Louis, MO). The peptides were dried in a MIVAC DUO vacuum evaporator (Genevac, Ipswich, UK) and dissolved in deionized distilled water/acetonitrile/trifluoroacetic acid (93:5:2). The samples were analyzed using nano-liquid chromatography-electrospray ionization-multi-stage mass spectrometry (LC-ESI-MS/MS) employing a model 1200 nano-flow system (Agilent Technologies, Palo Alto, CA) connected to a LTQ linear ion trap mass spectrometer (Thermo Electron, San Jose, CA). The reversed-phase capillary column was 12 cm in length, 75 mm in inner diameter and in-house packed with 5 μm 200 Å pore sized Magic C18AQ beads (Michrome BioResources, Auburn, CA).

The peptides were eluted in a linear gradient from 10 to 40% acetonitrile over 65 min. MS survey was scanned for 300-2000 *m/z* with three data-dependent MS/MS scans of isolation width of 1.5 *m/z*, normalized collision energy of 25%, and dynamic exclusion duration of 180 sec. MS data were generated in RAW file format (Thermo Scientific) using the Xcalibur1.4 with Tune1.0. Peptide peaks were introduced into MS/MS ions search in the Mascot server (http://www.matrixscience.com). Mass values were selected with monoisotopic masses. Peptide and MS/MS tolerances were ± 1.2 and ± 0.6 Da, respectively. To validate MS/MS results, Mascot files were loaded on Scaffold ver3.6 (Proteome Software, Portland, OR). Individual ions scores > 55 were considered significant or extensive homology (*P* < 0.05). In order to verify potential ambiguity between parasite- and host-derived proteins, we searched at least two peptides that were specific to either parasite or host. The proteins ambiguously assigned were excluded. We considered cysteine carbamidomethylation and methionine oxidation during the analyses. We independently analyzed each of the duplicated biological samples. We performed MS database search with merged files from non-redundant and expressed sequence tags on the NCBI database (https://www.ncbi.nlm.nih.gov/) and the *E. granulosus* and *E. multilocularis* gene and protein database of the Sanger Institute (http://www.genedb.org/Homepage).

Signal peptide and non-classical secretions were predicted by PSORT (http://psort.hgc.jp/), SignalP (http://www.cbs.dtu.dk/services/SignalP/), and the SecretomeP 2.0 server (http://www.cbs.dtu.dk/). A protein with *P* value of the D-score ≤ 0.05 was considered to possess a signal peptide. Non-classical pathways were defined when NN score was ≥ 0.5.

### Assignment of gene ontology (GO) terms

Functional classification of GO terms for the parasite and host proteins was carried out using Blast2GO ver4.0.7 (http://www.blast2go.com) with default parameters (cut-off of 30 for homology annotation) [19] according to the BLASTp searches of the identified proteins against the SwissProt (http://web.expasy.org/docs/swiss-prot_guideline.html) and NCBInr database. We subsequently conducted GO-mapping, InterProScan, and graphical analysis based on the BLASTp results. Histograms of biological process, molecular function, and cellular component were generated using the second level of the GO hierarchy.

### Analysis of functionally integrated protein-protein interaction (PPI) networks

We searched the EmHF and EgHF protein sequences for mouse homologs using Uniprot software (http://www.uniprot.org/) to predict functional PPI relationships [42]. We individually screened the UniProtKB entry numbers of the mouse homologs with the terms “multiple protein” and “*Mus musculus*” (Taxid: 10090). Subsequently, we entered the retrieved mouse homologs into the STRING ver10.0 database (http://string-db.org/) [44] for in silico mapping of putative physical and functional associations of EmHF and EgHF proteins. The average local clustering coefficient was 0.556 and the PPI enrichment *P*-value was 0. Each node and edge represented a protein and an interaction of pairwise proteins. We defined nodes with relatively large numbers of edges as hub proteins.

### Reverse-transcription PCR (RT-PCR)

Total RNAs (400 ng each) extracted from the AE mass (1, 3, 6, 9, and 14 months postinfection) and a single CE2 cyst were used for amplification of *E. multilocularis* NAALAD2 (*EmNAALAD2*; EmuJ_000908900) with a gene-specific forward primer (AACTGGAGAGATGTCTAC) and a reverse primer (CAGTGAACCAAGAAGTCC). RT-PCR cycling consisted of 30 min at 45°C and 5 min at 95°C, followed by 35 cycles of 1 min at 95°C, 45 sec at 50°C, 3 min at 72°C, and a final extension for 10 min at 72°C. PCR products were separated by 1% agarose gel electrophoresis and visualized by ethidium bromide staining. *EmActin* (EmuJ_000407400) was amplified as a positive control with gene-specific primers: forward, TCGAAGCGTGGTATTCTC and reverse, TTGAGTGGTGCCTCAGTT.

## SUPPLEMENTARY MATERIALS FIGURE AND TABLES




