# Supplementary material for: Comparison of Echinococcus multilocularis and Echinococcus granulosus hydatid fluid proteome provides molecular strategies for specialized host-parasite interactions
Source: Oncotarget. 2017 Sep 8;8(57):97009–24. doi: 10.18632/oncotarget.20761 (PMC5722541; doi:10.18632/oncotarget.20761)
Supplement: Supplementary file 1 [file oncotarget-08-97009-s001.pdf]

Comparison of *Echinococcus multilocularis* and *Echinococcus granulosus* hydatid fluid proteome provides molecular strategies for specialized host-parasite interactions

SUPPLEMENTARY MATERIALS

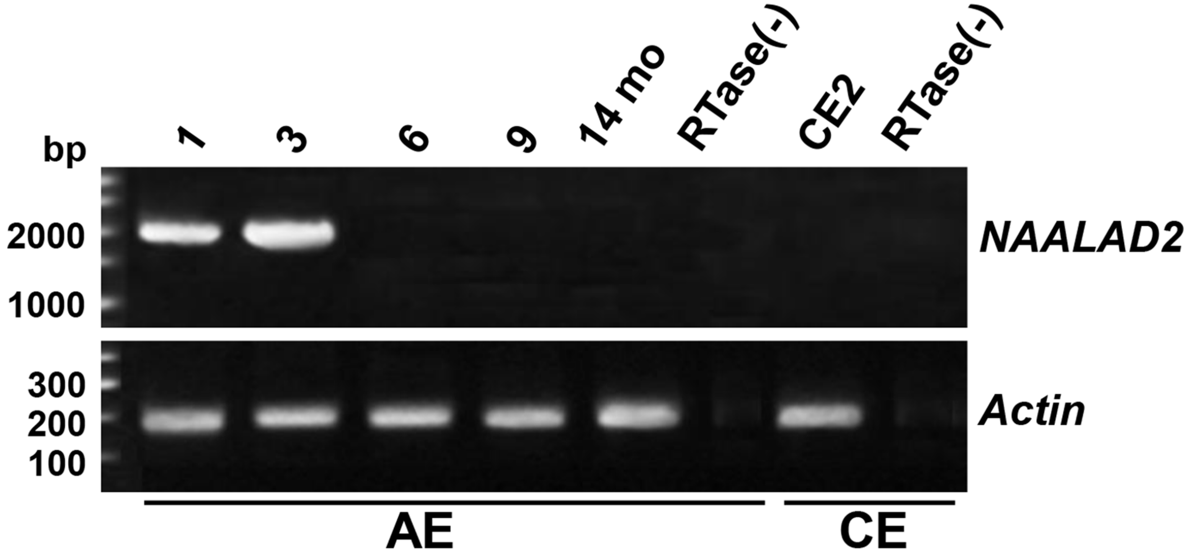

Supplementary Figure 1: Amplification of NAALAD2 gene by RT-PCR. The presence of NAALAD2 transcript was determined using gene-specific primers and total RNA extracted from 1, 3, 6, 9, and 14 month postinfections. RNA purified from a single CE2 cyst was also used for comparison. The *E. multilocularis* actin gene (EmuJ_000407400) was used as a control.

Supplementary Table 1: Identification of proteins in hydatid fluid of *E. multilocularis* metacestode by LC-ESI-MS/MS

See Supplementary File 1

Supplementary Table 2: Protein identifications in hydatid fluid of *E. granulosus* metacestode by LC-ESI-MS/MS

See Supplementary File 1

Supplementary Table 3: Host proteins found in both EmHF and EgHF (4 species)

No.	Band no.	Sum of emPAI[1]	Organism	Description	Accession no.[2]	Putative function
1	6-10, 13-22	10.36	Em	Albumin	BAE35818	Serum component
	16, 18	19.41	Eg		NP_001009376	
2	17-19	4.22	Em	Serotransferrin	AAH08559	Response to iron
	17, 18	2.57	Eg		W5PF65	
3	14, 15	0.62	Em	Serpin (α1 protease inhibitor)	AAA37132	Serine protease inhibitor
	14	0.1	Eg	Serpin A3-1	XP_011963751	
4	14	0.39	Em	Immunoglobulin	AAA51043	Antigen binding
	7, 15	0.21	Em	Ig-light chain	AAD47575	
	7	0.85	Eg	Ig lambda chain	B30554	

[1]Exponentially modified protein abundance index.
[2]Accession numbers were obtained from NCBInr DB (http://www.ncbi.nlm.nih.gov/).
Em, *E. multilocularis*; Eg, *E. granulosus*.

Supplementary Table 4: Host proteins solely identified in EmHF (8 species) or in EgHF (14 species)

Origin	No.	Band no.	Sum of emPAI[1]	Description	Accession no.[2]	Putative function
EmHF	1	5, 9, 10	0.15	mCG17605, isoform CRA_b	EDL04023	Structural protein
	2	6	0.13	Unnamed protein product	CAK96138	Not-available
	3	9	0.11	Phospholipid transfer protein	P55065	Lipid transport
	4	12	0.17	Actin	AAA37164	Cell growth
	5	14, 15	0.60	Fetuin (α-2HS-glycoprotein)	AAB81718	Cysteine protease inhibitor, phagocytosis
	6	16, 17	0.54	Contrapsin	CAA38948	Serine protease inhibitor
	7	16	0.07	Kininogen	AAH18158	Cysteine protease inhibitor
	8	17	0.09	Hemopexin	AAB49490	Metabolism, Signaling
EgHF	1	3	0.45	Fatty acid binding protein	XP_004005947	Fatty acid transport
	2	7	0.91	Glutathione transferase	CAB50870	Antioxidant
	3	7	0.16	Triosephosphate isomerase	W5P5W9	Glucose metabolism
	4	8	0.18	Carbonic anhydrase 2	P00922	pH regulation
	5	9	0.1	Interleukin-27 subunit beta	XP_004008649	Immune regulation
	6	10	0.32	Regucalcin	NP_001124407	Calcium binding
	7	10	0.19	Dihydrodiol dehydrogenase 3-like	C3SZI7	Amino acid metabolism
	8	11	0.09	Alcohol dehydrogenase 6-like	XP_004022883	Oxidoreductase
	9	12	1.04	Actin cytoplasmic type 1	P56401	Transporter
	10	13	0.06	Citrate synthase (mitochondrial)	XP_004006633	Carbohydrate metabolism
	11	14	0.19	Retinal dehydrogenase 1	P51977	Cofactor metabolism
	12	16	0.13	Aldehyde dehydrogenase family 8 member A1 isoform X2	W5Q9L8	Aldehyde metabolism
	13	19	0.14	Carbonic anhydrase inhibitor	XP_004003380	CA2 inhibitor
	14	3	0.28	Hemoglobin subunit beta	Q1KYZ7	Oxygen transport

[1]Exponentially modified protein abundance index.
[2]Accession numbers were obtained from NCBInr DB (http://www.ncbi.nlm.nih.gov/).
