# Supplementary material for: Comparison of Echinococcus multilocularis and Echinococcus granulosus hydatid fluid proteome provides molecular strategies for specialized host-parasite interactions
Source: Oncotarget. 2017 Sep 8;8(57):97009–24. doi: 10.18632/oncotarget.20761 (PMC5722541; doi:10.18632/oncotarget.20761)
Supplement: Supplementary file 2 [file oncotarget-08-97009-s002.docx]

**Supplementary Table 1: Identification of proteins in hydatid fluid of *E. multilocularis* metacestode by LC-ESI-MS/MS**

| **Band no.** | **MS^1^** | **emPAI** | **Organism** | **Description** | **Accession no.^2^ (EmuJ_)** | **SP** |
| --- | --- | --- | --- | --- | --- | --- |
| 1 | 161 | 1.47 | *Em* | EmAgB3b/c^3^ | 000381600/700 | + |
| 2 | 388 | 24.7 | *Em* | EmAgB3a | 000381500 | + |
|  | 386 | 3.19 | *Em* | EmAgB4 | 000381400 | + |
|  | 324 | 3.51 | *Em* | EmAgB1 | 000381200 | + |
|  | 319 | 21.51 | *Em* | EmAgB3b/c | 000381600/700 | + |
|  | 277 | 10.78 | *Em* | EmAgB2 | 000381100 | + |
| 3 | 206 | 1.42 | *Em* | EmAgB3a | 000381500 | + |
|  | 203 | 2.21 | *Em* | EmAgB3b/c | 000381600/700 | + |
|  | 95 | 0.33 | *Em* | EmAgB4 | 000381400 | + |
|  | 90 | 0.77 | *Em* | EmAgB2 | 000381100 | + |
| 4 | 254 | 3.37 | *Em* | EmAgB3a | 000381500 | + |
|  | 245 | 2.15 | *Em* | EmAgB4 | 000381400 | + |
|  | 201 | 2.15 | *Em* | EmAgB3b/c | 000381600/700 | + |
|  | 98 | 0.49 | *Em* | FABP2 | 000550000 | - |
|  | 95 | 0.49 | *Em* | Hedgehog | 001200300 | + |
|  | 72 | 0.49 | *Em* | Zinc finger, C2H2 | 000194800 | - |
| 5 | 140 | 0.05 | *Mm* | mCG17605 (isoform CRA_b) | EDL04023 | - |
| 6 | 103 | 0.05 | *Mm* | Albumin | BAE35818 | + |
|  | 89 | 0.05 | *Em* | Glycoprotein antigen 5 | 000184900 | + |
| 7 | 404 | 0.13 | *Em* | Collagen type XI α2 | 000524200 | + |
|  | 297 | 0.15 | *Mm* | Albumin | BAE35818 | + |
|  | 93 | 0.13 | *Em* | Glutathione transferase | 000538300 | - |
|  | 79 | 0.13 | *Mm* | Ig light chain | AAD47575 | - |
|  | 74 | 0.13 | *Mm* | Unnamed protein product | CAK96138 | + |
| 8 | 106 | 0.13 | *Mm* | Albumin | BAE35818 | + |
|  | 61 | 0.04 | *Em* | FGFR | 000842900 | - |
| 9 | 203 | 0.05 | *Mm* | mCG17605 (isoform CRA_b) | EDL04023 | - |
|  | 119 | 0.15 | *Mm* | Albumin | BAE35818 | + |
|  | 113 | 0.11 | *Em* | Proteinase inhibitor I25 | 000849600 | + |
|  | 60 | 0.11 | *Mm* | Phospholipid transfer protein | P55065 | + |
| 10 | 255 | 0.11 | *Mm* | Albumin | BAE35818 | + |
|  | 181 | 0.05 | *Mm* | mCG17605 (isoform CRA_b) | EDL04023 | - |
|  | 133 | 0.11 | *Em* | Proteinase inhibitor I25 | 000849600 | + |
|  | 126 | 0.19 | *Em* | Cytosolic malate dehydrogenase | 000417100 | - |
|  | 90 | 0.04 | *Em* | NAALAD2 | 000908900 | + |
|  | 63 | 0.01 | *Em* | α2 macroglobulin | 000641100 | + |
|  | 61 | 0.09 | *Em* | GAPDH | 000254600 | - |
| 11 | 411 | 0.49 | *Em* | FBA | 000905600 | - |
|  | 211 | 0.27 | *Em* | Cathepsin B | 000790200 | + |
|  | 124 | 0.04 | *Em* | Laminin | 000068100 | + |
|  | 112 | 0.08 | *Em* | NAALAD2 | 000908900 | + |
|  | 95 | 0.09 | *Em* | GAPDH | 000254600 | - |
|  | 74 | 0.01 | *Em* | α2 macroglobulin | 000641100 | + |
|  | 58 | 0.08 | *Em* | Annexin | 000193700 | - |
| 12 | 185 | 0.17 | *Em* | Cathepsin B | 000790200 | + |
|  | 164 | 0.17 | *Mm* | Actin | AAA37164 | - |
|  | 115 | 0.08 | *Em* | FBA | 000905600 | - |
|  | 103 | 0.03 | *Em* | LDLR | 000813700 | - |
|  | 98 | 0.02 | *Em* | Laminin | 000068100 | + |
| 13 | 964 | 1.05 | *Mm* | Albumin | BAE35818 | + |
|  | 437 | 0.5 | *Em* | Enolase | 000514200 | - |
|  | 58 | 0.5 | *Em* | α2 macroglobulin | 000641100 | + |
| 14 | 995 | 1.15 | *Mm* | Albumin | BAE35818 | + |
|  | 371 | 0.52 | *Em* | EP45 | 000824000 | - |
|  | 221 | 0.39 | *Mm* | Immunoglobulin | AAA51043 | + |
|  | 185 | 0.28 | *Mm* | Fetuin (α-2HS-glycoprotein) | AAB81718 | + |
|  | 174 | 0.3 | *Mm* | Serpin (α-1 protease inhibitor) | AAA37132 | + |
| 15 | 1364 | 1.26 | *Mm* | Albumin | BAE35818 | + |
|  | 328 | 0.32 | *Mm* | Serpin (α-1 protease inhibitor) | AAA37132 | + |
|  | 172 | 0.32 | *Mm* | Fetuin (α-2HS-glycoprotein) | AAB81718 | + |
|  | 141 | 0.08 | *Em* | NAALAD2 | 000908900 | + |
|  | 137 | 0.08 | *Em* | Phosphoglucose isomerase | 000626300 | - |
|  | 91 | 0.08 | *Em* | Glycoprotein antigen 5 | 000184900 | + |
|  | 90 | 0.08 | *Mm* | Immunoglobulin | AAA51043 | + |
| 16 | 1848 | 4.09 | *Mm* | Albumin | BAE35818 | + |
|  | 93 | 0.14 | *Mm* | Contrapsin | CAA38948 | + |
|  | 85 | 0.14 | *Em* | Gynecophoral canal protein | 000824400 | + |
|  | 66 | 0.07 | *Mm* | Kininogen | AAH18158 | + |
| 17 | 1390 | 1 | *Mm* | Serotransferrin | AAH08559 | - |
|  | 1113 | 1.48 | *Mm* | Albumin | BAE35818 | + |
|  | 356 | 0.4 | *Mm* | Contrapsin | CAA38948 | + |
|  | 241 | 0.1 | *Em* | LDLR | 000813700 | - |
|  | 157 | 0.08 | *Em* | NAALAD2 | 000908900 | + |
|  | 107 | 0.06 | *Mm* | Hemopexin | AAB49490 | + |
|  | 90 | 0.06 | *Em* | HSPG | 000575900 | + |
|  | 75 | 0.1 | *Em* | Diagnostic antigen gp50 | 001201600 | + |
|  | 74 | 0.1 | *Em* | Conserved hypothetical protein | 001058700 | - |
| 18 | 2052 | 3.07 | *Mm* | Serotransferrin | AAH08559 | - |
|  | 1052 | 1.05 | *Mm* | Albumin | BAE35818 | + |
|  | 1040 | 0.63 | *Em* | Gynecophoral canal protein | 000712600 | - |
|  | 835 | 0.63 | *Em* | NAALAD2 | 000908900 | + |
|  | 223 | 0.14 | *Em* | LDLR | 000813700 | - |
|  | 210 | 0.03 | *Em* | α2 macroglobulin | 000641100 | + |
|  | 132 | 0.08 | *Em* | Amiloride sensitive amine oxidase | 000530400 | + |
|  | 123 | 0.04 | *Em* | ADAMTS protein 3 | 000969100 | - |
|  | 59 | 0.03 | *Em* | Prosaposin a preproprotein | 000733100 | + |
|  | 58 | 0.03 | *Em* | Tyrosine protein kinase otk | 000212300 | + |
| 19 | 1010 | 1.05 | *Em* | NAALAD2 | 000908900 | + |
|  | 574 | 0.44 | *Mm* | Albumin | BAE35818 | + |
|  | 189 | 0.15 | *Mm* | Serotransferrin | AAH08559 | - |
|  | 182 | 0.03 | *Em* | α2 macroglobulin | 000641100 | + |
|  | 98 | 0.03 | *Em* | Fibrillar collagen chain FAp1α | 001060700 | - |
|  | 89 | 0.04 | *Em* | Laminin | 000068100 | + |
|  | 73 | 0.03 | *Em* | Lysosomal α glucosidase | 000141000 | + |
|  | 63 | 0.02 | *Em* | Collagen α2(I) chain | 000823800 | + |
| 20 | 244 | 0.16 | *Em* | NAALAD2 | 000908900 | + |
|  | 145 | 0.15 | *Mm* | Albumin | BAE35818 | + |
|  | 83 | 0.07 | *Em* | LDLR | 000813700 | - |
| 21 | 385 | 0.07 | *Em* | α2 macroglobulin | 000641100 | + |
|  | 360 | 0.07 | *Mm* | Albumin | BAE35818 | + |
|  | 279 | 0.08 | *Em* | Prosaposin a preproprotein | 000733100 | + |
|  | 137 | 0.08 | *Em* | NAALAD2 | 000908900 | + |
|  | 103 | 0.08 | *Em* | HSPG | 000575900 | + |
|  | 89 | 0.03 | *Em* | Fibrillar collagen FAp1α | 001060700 | - |
|  | 87 | 0.03 | *Em* | Neutral α glucosidase AB | 000716600 | + |
|  | 73 | 0.07 | *Em* | LDLR | 000813700 | - |
|  | 70 | 0.07 | *Em* | Lysosomal α mannosidase | 000704400 | + |
|  | 61 | 0.02 | *Em* | Collagen α1(V) chain | 000140100 | - |
| 22 | 2094 | 0.11 | *Em* | HSPG | 000575900 | + |
|  | 1517 | 0.34 | *Em* | α2 macroglobulin | 000641100 | + |
|  | 655 | 0.44 | *Em* | LDLR | 000813700 | - |
|  | 369 | 0.09 | *Em* | Collagen α1(V) chain | 000140100 | - |
|  | 362 | 0.08 | *Em* | Collagen α1(IV) chain | 000140000 | + |
|  | 327 | 0.08 | *Mm* | Albumin | BAE35818 | + |
|  | 289 | 0.25 | *Em* | NAALAD2 | 000908900 | + |
|  | 172 | 0.04 | *Em* | Peroxidasin | 000733600 | + |
|  | 158 | 0.06 | *Em* | Emb9 | 000139900 | + |
|  | 79 | 0.11 | *Em* | Neurogenic locus notch protein | 000343000 | - |
|  | 69 | 0.11 | *Em* | EGF domain protein | 000255800 | + |
|  | 65 | 0.01 | *Em* | Prosaposin a preproprotein | 000733100 | + |

^1^Mascot score: protein scores greater than 55 are significant and individual ions scores > 34 indicate identical or extensive homology (*P* < 0.05).

^2^Accession numbers were obtained from NCBInr DB (<http://www.ncbi.nlm.nih.gov/>) and *E. multilocularis* DB (<http://www.genedb.org/Homepage/Emultilocularis>).

^3^EmAgB3a and EmAgB3b/c identification was tailored by Ahn et al. [16].

emPAI, exponentially modified protein abundance index; *Em*, *Echinococcus multilocularis*; Emb9, abnormal EMBroylocus tagsis emb 9; EP45, estrogen regulated protein EP45; FABP2, fatty acid binding protein 2; FBA, fructose bisphosphate aldolase; FGFR, fibroblastic growth factor receptor; GAPDH, glyceraldehyde 3-phosphate dehydrogenase; HSPG, basement membrane specific heparan sulfate; LDLR, low-density lipoprotein receptor; *Mm*, *Mus musculus;* NAALAD2, N-acetylated alpha-linked acidic dipeptidase 2; SP, signal peptide.

**Supplementary Table 2: Protein identifications in hydatid fluid of *E. granulosus* metacestode by LC-ESI-MS/MS**

| **Band no.** | **MS^1^** | **emPAI** | **Organism** | **Description** | **Accession no.^2^ (EgrG_)** | **SP** |
| --- | --- | --- | --- | --- | --- | --- |
| 2 | 1031 | 20.61 | *Eg* | EgAgB1 | 000381200 | + |
|  | 417 | 5.86 | *Eg* | EgAgB4 | 000381400 | + |
|  | 396 | 3.25 | *Eg* | EgAgB2 | 000381100 | + |
|  | 159 | 4.57 | *Eg* | EgAgB3 | 000381600 | + |
| 3 | 258 | 1.38 | *Eg* | EgAgB2 | 000381100 | + |
|  | 227 | 1.62 | *Eg* | EgAgB4 | 000381400 | + |
|  | 122 | 0.28 | *Oa* | Hemoglobin subunit β | NP_001091117 | - |
|  | 95 | 2.95 | *Eg* | EgAgB3 | 000381600 | + |
|  | 92 | 5.83 | *Eg* | EgAgB1 | 000381200 | + |
|  | 90 | 0.21 | *Eg* | Expressed conserved protein | 000378300 | - |
|  | 77 | 0.45 | *Oa* | FABP | XP_004005947 | - |
|  | 69 | 0.33 | *Oa* | α globin chain | CAA49750 | - |
|  | 57 | 0.28 | *Eg* | Expressed conserved protein | 000956500 | + |
|  | 55 | 0.22 | *Eg* | Superoxide dismutase 1 soluble | 000638300 | - |
|  | 53 | 0.26 | *Eg* | Profilin allergen | 000122100 | - |
| 4 | 735 | 12.04 | *Eg* | EgAgB4 | 000381400 | + |
|  | 330 | 3.89 | *Eg* | EgAgB2 | 000381100 | + |
|  | 137 | 1.86 | *Eg* | Expressed conserved protein | 000079500 | + |
|  | 122 | 4.86 | *Eg* | EgAgB1 | 000381200 | + |
|  | 83 | 2.95 | *Eg* | EgAgB3 | 000381600 | + |
|  | 77 | 0.21 | *Eg* | Expressed conserved protein | 000378300 | - |
| 5 | 626 | 4.86 | *Eg* | Glycoprotein antigen 5 | 000184900 | + |
|  | 442 | 5.86 | *Eg* | EgAgB4 | 000381400 | + |
|  | 215 | 0.78 | *Eg* | EgAgB2 | 000381100 | + |
|  | 116 | 0.88 | *Eg* | Ferritin | 000382200 | - |
|  | 84 | 0.2 | *Eg* | U snRNP-associated cyclophilin protein | 000525910 | - |
|  | 83 | 4.86 | *Eg* | EgAgB1 | 000381200 | + |
|  | 62 | 0.21 | *Eg* | Expressed conserved protein | 000378300 | - |
|  | 58 | 0.99 | *Eg* | EgAgB3 | 000381600 | + |
| 6 | 2013 | 10.14 | *Eg* | Glycoprotein antigen 5 | 000184900 | + |
|  | 568 | 11.68 | *Eg* | EgAgB4 | 000381400 | + |
|  | 390 | 2.08 | *Eg* | Expressed conserved protein | 000596300 | + |
|  | 254 | 0.78 | *Eg* | EgAgB2 | 000381100 | + |
|  | 154 | 1.29 | *Eg* | Expressed protein | 000315600 | + |
|  | 128 | 4.86 | *Eg* | EgAgB1 | 000381200 | + |
|  | 88 | 0.74 | *Eg* | Ferritin | 000382200 | - |
|  | 80 | 0.25 | *Eg* | Peptidase inhibitor 16 | 000766600 | + |
|  | 78 | 0.35 | *Eg* | Thioredoxin peroxidase | 000791700 | - |
|  | 73 | 0.99 | *Eg* | EgAgB3 | 000381600 | + |
| 7 | 433 | 3.98 | *Eg* | EgAgB4 | 000381400 | + |
|  | 177 | 1.59 | *Eg* | EgAgB2 | 000381100 | + |
|  | 162 | 0.85 | *Oa* | Ig lambda chain C region | B30554 | - |
|  | 105 | 0.25 | *Eg* | Peptidase inhibitor 16 | 000766600 | + |
|  | 96 | 0.16 | *Eg* | Niemann Pick C2 protein | 000682900 | + |
|  | 75 | 0.91 | *Oa* | Glutathione S-transferase | CAB50870 | - |
|  | 65 | 0.13 | *Eg* | Complement C1q tumor necrosis factor | 001189200 | + |
|  | 62 | 0.16 | *Oa* | Triosephosphate isomerase | XP_011984860 | - |
|  | 57 | 0.12 | *Eg* | Triosephosphate isomerase | 000416400 | - |
|  | 46 | 0.56 | *Eg* | EgAgB1 | 000381200 | + |
| 8 | 160 | 1.62 | *Eg* | EgAgB4 | 000381400 | + |
|  | 150 | 0.25 | *Eg* | Peptidase inhibitor 16 | 000766600 | + |
|  | 96 | 0.57 | *Eg* | 14-3-3 protein | 000789700 | - |
|  | 89 | 0.89 | *Eg* | EgAgB2 | 000381100 | + |
|  | 79 | 0.26 | *Eg* | Protein l isoaspartate o methyltransferase | 001133400 | - |
|  | 76 | 0.1 | *Eg* | Phosphoglycerate mutase | 000799500 | - |
|  | 59 | 0.18 | *Oa* | Carbonic anhydrase 2 | P00922 | - |
|  | 52 | 0.2 | *Eg* | Endophilin B1 | 000550800 | - |
|  | 41 | 0.39 | *Eg* | EgAgB1 | 000381200 | + |
| 9 | 373 | 0.85 | *Eg* | Proteinase inhibitor I25 | 000849600 | + |
|  | 247 | 3.98 | *Eg* | EgAgB4 | 000381400 | + |
|  | 186 | 0.78 | *Eg* | EgAgB2 | 000381100 | + |
|  | 119 | 0.55 | *Eg* | Peptidase inhibitor 16 | 000766600 | + |
|  | 92 | 0.65 | *Eg* | EgAgB1 | 000381200 | + |
|  | 48 | 0.41 | *Eg* | EgAgB3 | 000381600 | + |
|  | 43 | 0.1 | *Oa* | Interleukin-27 subunit beta | XP_004008649 | + |
| 10 | 1934 | 3.05 | *Eg* | Glycoprotein antigen 5 | 000184900 | + |
|  | 271 | 1.44 | *Eg* | Malate dehydrogenase | 001185000 | - |
|  | 208 | 0.51 | *Eg* | Proteinase inhibitor I25 | 000849600 | + |
|  | 204 | 2.61 | *Eg* | EgAgB4 | 000381400 | + |
|  | 156 | 0.31 | *Eg* | Lactate dehydrogenase A | 000660800 | - |
|  | 139 | 0.89 | *Eg* | EgAgB2 | 000381100 | + |
|  | 132 | 0.32 | *Oa* | Regucalcin | NP_001124407 | - |
|  | 114 | 0.2 | *Eg* | Succinyl CoA | 001199000 | - |
|  | 106 | 0.68 | *Eg* | Cytosolic malate dehydrogenase | 000417100 | - |
|  | 99 | 0.23 | *Eg* | Insulin growth factor binding | 000799300 | + |
|  | 93 | 0.55 | *Eg* | GAPDH | 000254600 | - |
|  | 88 | 0.19 | *Oa* | Dihydrodiol dehydrogenase 3-like | XP_012044016 | - |
|  | 71 | 0.29 | *Eg* | Transaldolase | 000092800 | - |
|  | 63 | 0.12 | *Eg* | Peptidase inhibitor 16 | 000766600 | + |
|  | 62 | 0.17 | *Eg* | FBA | 000905600 | - |
|  | 60 | 0.08 | *Eg* | Pyruvate dehydrogenase | 000956200 | - |
|  | 49 | 0.94 | *Eg* | EgAgB1 | 000381200 | + |
| 11 | 421 | 0.59 | *Eg* | Glycoprotein antigen 5 | 000184900 | + |
|  | 238 | 1.42 | *Eg* | FBA | 000905600 | - |
|  | 230 | 0.92 | *Eg* | Cathepsin B | 000790200 | + |
|  | 219 | 1.62 | *Eg* | EgAgB4 | 000381400 | + |
|  | 111 | 0.78 | *Eg* | EgAgB1 | 000381200 | + |
|  | 86 | 0.2 | *Eg* | Succinyl CoA | 001199000 | - |
|  | 72 | 0.09 | *Oa* | Alcohol dehydrogenase 6-like | XP_004022883 | - |
|  | 58 | 0.08 | *Eg* | Malate dehydrogenase | 001185000 | - |
| 12 | 272 | 1.04 | *Eg* | Actin cytoplasmic type 5 | 000190400 | - |
|  | 267 | 1.15 | *Eg* | Ornithine aminotransferase | 001032200 | - |
|  | 246 | 2.61 | *Eg* | EgAgB4 | 000381400 | + |
|  | 215 | 3.59 | *Oa* | Actin, cytoplasmic 1 | P56401 | - |
|  | 179 | 0.77 | *Eg* | Cathepsin B | 000790200 | + |
|  | 149 | 0.41 | *Eg* | Enolase | 000514200 | - |
|  | 125 | 0.54 | *Eg* | EP45 | 000824000 | - |
|  | 63 | 0.27 | *Eg* | FBA | 000905600 | - |
| 13 | 565 | 1.54 | *Eg* | Citrate synthase | 001028500 | - |
|  | 242 | 0.5 | *Eg* | Glycoprotein antigen 5 | 000184900 | + |
|  | 152 | 0.4 | *Eg* | NADPH | 001068500 | - |
|  | 108 | 0.89 | *Eg* | EgAgB2 | 000381100 | + |
|  | 96 | 0.25 | *Eg* | PDHE1 | 000590700 | - |
|  | 57 | 0.06 | *Oa* | Citrate synthase (mitochondrial) | XP_004006633 | - |
|  | 51 | 0.12 | *Eg* | Acetyl CoA H/T | 001087900 | - |
| 14 | 190 | 0.5 | *Eg* | Glycoprotein antigen 5 | 000184900 | + |
|  | 183 | 1.62 | *Eg* | EgAgB4 | 000381400 | + |
|  | 149 | 0.64 | *Eg* | Calnexin | 000875100 | + |
|  | 80 | 0.19 | *Oa* | Retinal dehydrogenase 1 | NP_001009778 | - |
|  | 78 | 0.1 | *Oa* | Serpin A3-1-like | XP_011963751 | + |
|  | 72 | 0.27 | *Eg* | Iron:zinc purple acid phosphatase protein | 001169400 | + |
|  | 64 | 0.12 | *Eg* | Glutamate dehydrogenase | 000589100 | - |
| 15 | 248 | 0.68 | *Eg* | Phosphoglucose isomerase | 000626300 | - |
|  | 162 | 0.57 | *Eg* | Calnexin | 000875100 | + |
|  | 139 | 0.14 | *Eg* | Gynecophoral canal protein | 000824400 | + |
|  | 67 | 0.89 | *Eg* | EgAgB2 | 000381100 | + |
| 16 | 1996 | 17.51 | *Oa* | Serum albumin | NP_001009376 | + |
|  | 628 | 1.33 | *Eg* | Vesicular amine transporter | 000317300 | - |
|  | 327 | 0.34 | *Eg* | Glycoprotein antigen 5 | 000184900 | + |
|  | 144 | 0.38 | *Eg* | EgAgB4 | 000381400 | + |
|  | 92 | 0.06 | *Eg* | Lysyl oxidase | 000217900 | + |
|  | 65 | 0.12 | *Eg* | Aspartyl tRNA synthetase cytoplasmic | 000777100 | - |
|  | 51 | 0.13 | *Oa* | ADH8A1 | XP_004011403 | - |
| 17 | 592 | 1.07 | *Eg* | PEPCK | 000292700 | - |
|  | 376 | 0.93 | *Eg* | Gynecophoral canal protein | 000824400 | + |
|  | 166 | 1.93 | *Oa* | Serotransferrin | XP_011958592 | - |
|  | 80 | 0.08 | *Eg* | Amiloride sensitive amine oxidase | 000530400 | + |
|  | 64 | 0.08 | *Eg* | ADAMTS protein 3 | 000969100 | - |
| 18 | 250 | 0.37 | *Eg* | Lysosomal α mannosidase | 000704400 | + |
|  | 199 | 0.39 | *Eg* | ADAMTS protein 3 | 000969100 | - |
|  | 175 | 1.9 | *Oa* | Serum albumin | NP_001009376 | + |
|  | 148 | 0.26 | *Eg* | Glycoprotein antigen 5 | 000184900 | + |
|  | 127 | 0.38 | *Eg* | EgAgB4 | 000381400 | + |
|  | 85 | 0.34 | *Eg* | EgAgB1 | 000381200 | + |
|  | 76 | 0.64 | *Oa* | Serotransferrin | XP_011958592 | - |
| 19 | 191 | 0.31 | *Eg* | Amiloride sensitive amine oxidase | 000530400 | + |
|  | 76 | 0.14 | *Eg* | PEPCK | 000292700 | - |
|  | 65 | 0.14 | *Oa* | Inhibitor, carbonic anhydrase | XP_004003380 | - |
| 20 | 126 | 0.85 | *Eg* | Gynecophoral canal protein | 000712600 | - |
|  | 81 | 0.07 | *Eg* | Macroglobulin:complement | 000641200 | - |
| 21 | 423 | 1.04 | *Eg* | Gynecophoral canal protein | 000712600 | - |
|  | 170 | 0.31 | *Eg* | HSPG | 000575900 | + |
|  | 162 | 0.15 | *Eg* | Prosaposin a preproprotein | 000733100 | + |
|  | 101 | 0.05 | *Eg* | Collagen α1(XV) chain | 000729300 | - |
|  | 59 | 0.1 | *Eg* | Lysosomal α glucosidase | 000143500 | + |
|  | 50 | 0.09 | *Eg* | Peroxidasin | 000733600 | + |
| 22 | 562 | 0.23 | *Eg* | EGF domain protein | 000255800 | + |
|  | 494 | 0.2 | *Eg* | Neurogenic locus notch protein | 000343000 | - |
|  | 492 | 0.12 | *Eg* | HPSG | 000575900 | + |
|  | 270 | 0.08 | *Eg* | Collagen α1(IV) chain | 000144350 | + |
|  | 145 | 0.13 | *Eg* | Laminin | 000068100 | + |
|  | 132 | 0.08 | *Eg* | Emb9 | 000144400 | + |
|  | 127 | 0.04 | *Eg* | Hemicentin 1 | 000422350 | + |

^1^Mascot score; protein scores greater than 55 are significant and individual ions scores > 34 indicate identical or extensive homology (*P* < 0.05).

^2^Accession numbers were obtained from NCBInr DB (<http://www.ncbi.nlm.nih.gov/>) and *E. granulosus* DB (<http://www.genedb.org/Homepage/Egranulosus>).

Acetyl CoA HT, acyl coenzyme A hydrolase transferase; ADH8A1, aldehyde dehydrogenase family 8 member A1 isoform 2; *Eg*, *Echinococcus granulosus*; Emb9, abnormal embryogenesis family member emb 9; emPAI, exponentially modified protein abundance index; FBA, fructose bisphosphate aldolase; GAPDH, glyceraldehyde 3-phosphate dehydrogenase; HSPG, basement membrane specific heparan sulfate; NADPH, NADP-dependent isocitrate dehydrogenase; *Oa*, *Ovis aries*; PDHE1, pyruvate dehydrogenase E1 component subunit; PEPCK, phosphoenolpyruvate carboxykinase; SP, signal peptide; Succinyl CoA, succinyl coenzyme A synthetase α subunit.
